# A Point as to the Use of Tuberculin

**Published:** 1911-07

**Authors:** 


### A Point as to the Use of Tuberculin.

White and Van Norman in the *Archives of Internal Medicine* of October 15, 1910, say that the question of lymphatic distribution is a point which must be borne very strictly in mind in treatment with tuberculin, the focal reaction around the tuberculous lesion being a requisite factor in treatment. The dose in treatment may be so given as to drain along the lymphatic into the site of the lesion, and thus obtain the benign influence of focal reaction. This would apply to glands, sinuses, bone lesions and lupus.—*Therapeutic Gazette.*

